# Analysis of machete cut fractures in Nigerian civilian trauma setting

**DOI:** 10.1038/s41598-020-79981-w

**Published:** 2021-01-08

**Authors:** Njoku Isaac Omoke, Omolade Ayoola Lasebikan, Francis Ndubuisi Ahaotu, Ugochukwu Uzodimma Nnadozie, Gregory Chinedu Nwigwe

**Affiliations:** 1 Department of Surgery, Ebonyi State University/Federal Teaching Hospital, Abakaliki, Ebonyi State Nigeria; 2 National Orthopaedic Hospital, Enugu, Enugu State Nigeria; 3 Department of Surgery, Federal Teaching Hospital, Abakaliki, Ebonyi State Nigeria

**Keywords:** Health care, Medical research

## Abstract

Machete cut fracture is an important component of morbidity associated with machete injuries although it is under reported. This was a retrospective study to assess machete cut fractures in patients seen in Federal Teaching Hospital Abakaliki and National Orthopaedic Hospital Enugu from 2009 to 2018. There were 91 patients with 154 fractures, male- to- female ratio was 10:1 and mean age was 31.6 ± 14.6 years. The aetiological factors were assault (57, 62.6%), armed robbery (29, 31.9%) and accidental injury (5, 5.5%). The three top bones involved were ulna, metacarpal and finger-phalanx. Fracture was communited in (17, 11.0%), and Gustilo Anderson grade IIIC in (22, 14.3%). Injury to hospital arrival interval later than 6 h was common and correlated with prolonged length of hospital stay (p < 0.001). Anaemia, wound infection and hemorrhagic shock were the three top complications. Nine (5.8%) fractures ended in extremity amputation. Eleven (12.1%) patients left against medical advice, and 5 (5.5%) were transferred. Normal union in 98.3% of the fractures treated and followed up for a minimum of one year. Case fatality rate was 2.2%; none of the patient that died had pre hospital care, and hemorrhagic shock accounted for all the mortality. These call for appropriate injury preventive mechanisms, and improved rates of early presentation of patients to hospital, and pre hospital care.

## Introduction

Machete, a long knife with a sharp edged broad blade, is a common domestic and farm tool as well as a weapon in game hunting and assault in developing countries^1^. Machete varies in weight, rigidity and sharpness. In most African countries, the sharp, rigid, and relatively heavy machete is easily obtainable on demand and readily available in many homes; there is no legislation guiding its acquisition and use^1^. Machete wielded either as a farming tool or weapon of assault can cause accidental or intentional wounds of varying degrees of severity depending on the mechanism of injury, weight, rigidity and sharpness of the machete. A blow with the long cutting edge of the blade, the most frequent mechanism of injury, has the potential to slice through soft tissues as well as the underlying bone^1,2^. The potential to slice through bones is higher with sharp, rigid and relatively heavy machetes compared to blunt and springy ones^3^. Thus, partial or complete break in structural continuity of bone by machete cut is invariably an open fracture. In a recent published report, machete cut fracture accounted for 9% of open extremity fractures seen in a Nigerian tertiary hospital emergency room setting^4^. It is an important component of morbidity associated with machete injuries in developing countries where published reports indicate that one in every five to six victims of machete cut is at risk of an associated open fracture^1,5^.

Machete cut results in focal and guillotine type of injury usually with great depth and little or no evidence of contusion^3^. Thus, machete cut fracture wound may seem trivial compared to other open fracture wounds of high energy and crushing mechanism of injury^3^. The attending surgeon should be aware of potential damage to neurovascular bundles, tendons and muscles in the depth of the wound, which have implications in the severity, complexity, treatment strategies and outcome of machete related fractures^1,2^. Machete cut fracture being an open one is also prone to risk of infection and other wound complications^6^. However, the risk of infection is relatively low compared to open fractures resulting from high energy and crushing mechanisms of injury^3,7,8^. The management of machete-cut fracture usually entails a multidisciplinary workforce and can be quite challenging especially in a setting where there is paucity of centres with capacity to provide adequate care, as often the case in developing countries. A good knowledge of pattern and outcome of machete cut fracture can facilitate preventive strategies and intervention aimed at achieving optimum care of the victims. However, there is very scanty data on machete cut fractures; the ones available in literature are either anecdotal case reports or case series that focused on machete cut fractures involving specific anatomical region of the body^7,9–12^. Thus, paucity of data on machete cut fractures necessitated this study. The aim of this study was to determine the pattern and outcome of machete cut fractures seen in civilian trauma setting of a developing country.

## Methods

This was a retrospective analysis of data on all the consecutive patients that presented with machete cut fracture at the Emergency room of two Nigerian tertiary hospitals, Federal Teaching Hospital Abakaliki Ebonyi State and National Orthopaedic Hospital Enugu from 1st January 2009 to 31st December 2018. Federal Teaching hospital Abakaliki and is one of the University teaching hospitals Southeast Nigeria. National Orthopaedic Hospital Enugu is a regional orthopaedic and trauma centre located in Enugu southeast Nigeria. These two centres serve the Southeast, South-South and part of the North-Central geopolitical zones of Nigeria.

All the cases of machete cut fractures seen in the hospitals within the period were identified from the hospital admission database and the medical record folders of these patients retrieved. Information extracted from these case notes included: demographic data (age, gender, marital status and occupation), aetiology of injury, location (rural/urban), season (dry/wet) and immediate location injury (outdoor/indoor). The interval between injury and presentation to the hospital, pre-hospital care, co-morbidities, associated injuries and treatment, length of hospital stay, outcome and complications, were also extracted from the patient case notes. A serial coordinated multidisciplinary team approach involving emergency physicians, surgeons (orthopaedic, plastic and reconstructive, vascular, maxillofacial and neurosurgeon), anesthetist, radiologist, nurses, physiotherapist and others as the case demanded was the modality of care in all the patients. Patients presenting to the hospitals with machete cut were admitted in the Accident and Emergency (A&E) unit for resuscitation using the Advanced Trauma Life Support (ATLS) protocol, initial wound care, and administration of empirical parenteral antibiotics and tetanus toxoid. After this initial wound care, patients with contaminated lacerations, underwent wound exploration ± judicious debridement then wound irrigation with adequate amount of normal saline. Apparently clean wounds presenting within six hours of injury were closed primarily. Wound presenting later than 6 h or grossly contaminated was left open and inspected after 48 h; thereafter it was either closed or dressed daily in the surgical ward with normal saline and povidone iodine until it was clean and good enough for delayed primary closure/secondary closure or split skin grafting. The method of immobilization of fractures was dependent on multiple factors that included type of bone involved, the type and grade of open fracture and associated soft tissue injury. POP slab/cast was used for partial fractures, undisplaced fractures and after manipulation of some stable displaced fractures. Other conservative modes of immobilization were firm bandaging, collar and cuff and broad arm sling. Emergency/primary external fixation and percutaneous Kirschner wire fixation, and inter dental wire fixation were applied when indicated. All the internal fixation of fractures with plate and screws, intramedullary nail, and cerclage wires were delayed until there was no clinical and microbiological evidence of wound contamination/infection. Data was analyzed using IBM Statistical package for Social Sciences, IBM SPSS Statistics version 20 (IBM Corp. Armonk, N.Y. USA) statistical soft ware for graphs, frequency tables and cross tabulation. Chi squared test was used for statistical test of significance and p value < 0.05 was considered significant.

Data collection and usage in this study was in accordance with the principles of Helsinki declaration. The study was approved by Research Ethics Committee of the Federal Teaching Hospital Abakaliki Ebonyi State Nigeria (FETHA/Rec/vol2/2019/229, protocol no 03/03/2019–12/03/2019). The need for written informed consent in a retrospective analysis of anonymised patient data was waived by the Research Ethics Committee that approved the study protocol.

## Results

Within the 10 year period, there were 154 machete cut fractures in 91 patients that presented in the Emergency Departments of Federal Teaching Hospital Abakaliki and National Orthopaedic Hospital Enugu. The male- to- female ratio was 10.4:1. The age ranged from 7 to 80 years with a mean of 31.6 ± 14.6 years. The peak age incidence was 18–39 years; students, farmers and traders were the three top occupations of the patients involved as shown in Table 1.

**Table 1:** Aetiology of machete cut fractures by demographic characteristics and location.

	Aetiology			Total (%)	*P* value
	Accidental (%)	Armed robbery (%)	Assault (%)		
**Age (years)**					
0–17	3 (37.5)	1 (12.5)	4 (50.0)	8 (8.8)	0.002
18–39	1 (1.6)	21 (33.3)	41 (65.1)	63(69.2)	
40–65	0 (0.0)	6 (42.9)	8 (51.1)	14 (15.5)	
> 65	1 ((16.7)	1 (16.7)	4 (66.7)	6 (6.7)	
**Sex**					
Male	5 (6.0)	25 (30.1)	53 (63.9)	83 (91.2)	0.447
Female	0 (0.0)	4 (50.0)	4 (50.0)	8 (8.8)	
**Occupation**					
Students	3 (11.1)	3 (11.1)	21 (77.8)	27 ( 29.7)	0.029
Farmers	1 (4.3)	6 (26.1)	16 (69.6)	23 (25.3)	
Traders	1 (6.7)	6 (40.0)	8 (53.3)	15 (16.5)	
Artisan	0 (0.0)	1 (12.5)	7 (87.5)	8 (8.8)	
Drivers	0 (0.0)	7 (87.5)	1 (12.0)	8 (8.8)	
Civil servants	0 (0.0)	3 (60.0)	2 (40.0)	5 (5.5)	
Police/security agents	0 (0.0)	2 (50.0)	2 (50.0)	4 (4.4)	
House wives	0 (0.0)	1 (100.0)	0 (0.0)	1 (1.1)	
**Location**					
Rural	3 (6.1)	12 (24.5)	34 (69.4)	49 (53.8)	0.264
Urban	2 (4.8)	17 (40.5)	23 (54.8)	42 (46.2)	

The aetiological factors for machete cut fractures were accidental injury (5, 5.5%), armed robbery (29, 31.9%) and assault (57, 62.6%). Of the 57 assault related machete cut fractures, secret cultism (16, 28.7%), interpersonal violence/fight (15, 26.7%), domestic violence (8, 14.0%), mob action (5, 8.8%), vigilante action (4, 7.0%), political motivated violence (3, 5.3%), communal clashes (2, 3.5%), land disputes (2, 3.5%) and substance abuse (2, 3.5%) were involved. The highest incidence of accidental machete cut fracture was among children whereas the highest rate of armed robbery related machete cut fractures was among middle aged adults as shown in Table 1. Of the 5 patients with accidental machete cut fractures, 3 of them had it during farming activities whereas one victim had it while separating a fight and the other was an accidental injury from a machete wielded to kill a strange mad dog. All the 3 children involved in accidental injuries sustained fractures during farming related activities. Fifty three (58.2%) of the patients had the fracture in dry season whereas 38 (41.8%) had fracture in wet/rainy season. In forty nine (53.8%) of the patients, the fracture occurred in a rural area where as in 42 (46.2%) the fracture occurred in urban area. The incidence of assault related fracture was higher in rural than urban areas where as the rate of armed robbery related fractures was higher in the urban than rural areas as shown in Table 1.

Forty five patients (49.5%) had fractures involving ≥ 2 bones (multiply fractured patients) whereas 46 (50.5%) had fracture only in one bone. Of the 45 multiply fractured patients, assault, armed robbery and accidental injury was the aetiology in 24 (53.3%), 19 (42.2%) and 2(4.4%) of them respectively (p < 0.10). There was involvement of more than one phalanges of different fingers in same hand in two of the accident related multiply fractured patients. Fifty five (60.4%) of the patients sustained additional machete cut soft tissue injury in anatomical site different from the one of the fracture (additional distant soft tissue machete injury). The incidence of this additional distant soft tissue machete injury correlated (p < 0.009) with the aetiology of injury; the rate of this additional distant soft tissue injury was 72.4%, 59.6% and 0.0% in armed robbery, assault and accident related fractures respectively. Machete injuries to the scalp and upper extremity, irrespective of anatomical region of the bones involved, were observed in 45 (49.5%) of the patients.

**Figure 1:**
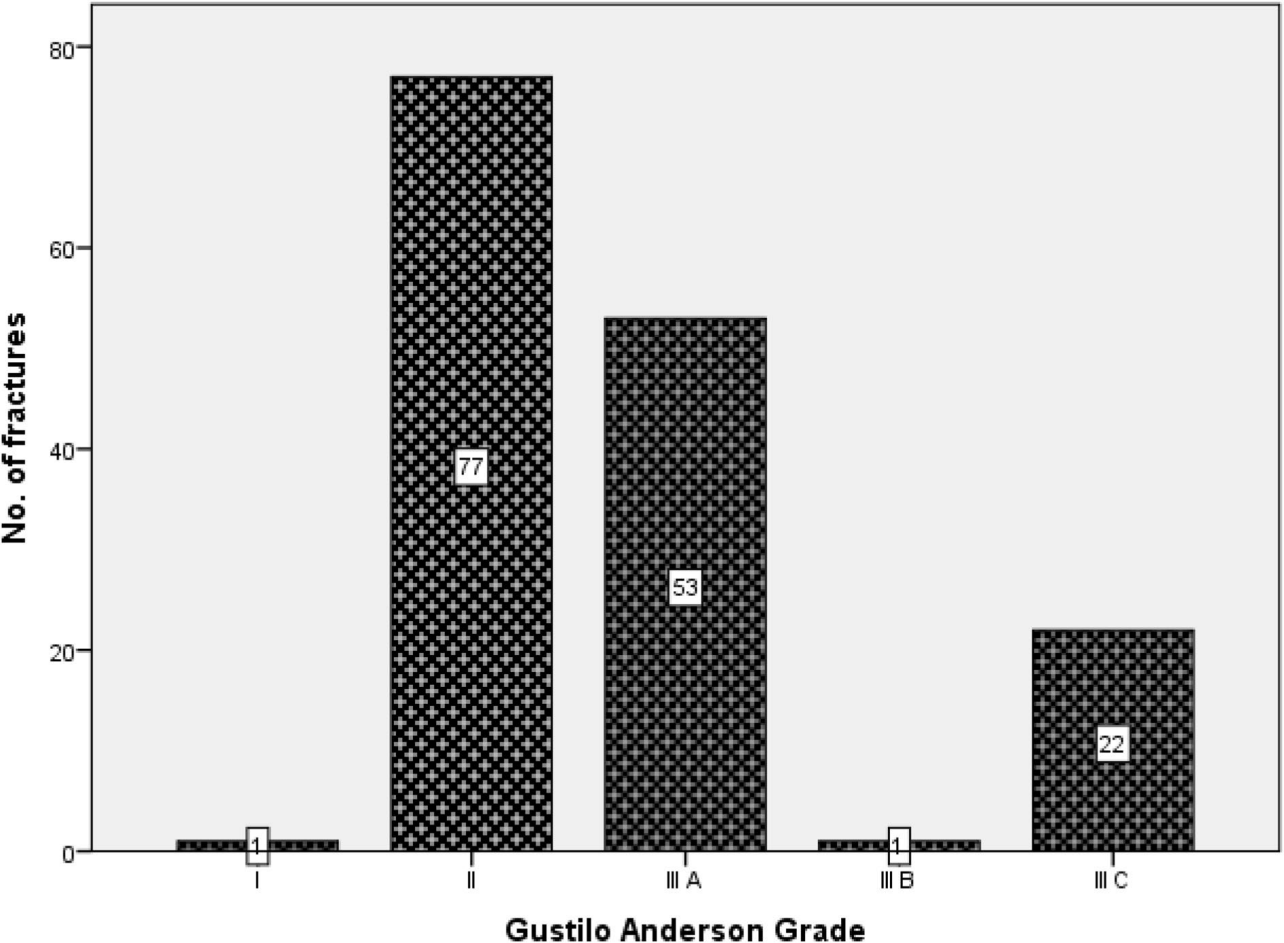
Distribution of 154 machete cut fractures by Anderson and Gustilo Classification. Figure designed using IBM Statistical package for Social Sciences, IBM SPSS Statistics version 20 (IBM Corp. Armonk, N.Y. USA) statistical soft ware https://www.ibm.com/products/spss-statistics.

Of the 154 fracture, 129 (83.8%) were complete fractures and 25 (16.2%) were partial (fissures, unicortical) fractures. The fracture line morphology observed were transverse (86, 55.8%), oblique (47, 30.5%), communited (17, 11.0%) and segmental (4, 2.6%) fractures. Machete cut to bones, in the upper extremity and lower extremities accounted for 69.5% and 20.3% of all the fractures respectively. In Table 2, the topmost bone involved among the female victims was the finger phalanx and among the male victims was ulna. Overall the three top bones involved were ulna, finger- phalanx and metacarpal as also shown in Table 2. The estimated open wound length was in range of 1 to 25 cm with a mean of 7.3 ± 4.4 cm. Gustilo Anderson grade II in 77 (50%) was the predominant type of machete cut open fracture as shown in Fig. 1. Extensor tendon of the hand, radial and ulna nerves, and radial artery were the top associated tendon, nerves and vascular injuries respectively as shown in Table 3. These associated tendon and neurovascular injuries were all guillotine type of injury.

**Table 2:** Distribution of 154 machete-cut fractures by gender and bone involved.

Bone	Gender		Total (%)	
	Male	Female		
Cranial vault	12	1	13	8.4
Maxillofacial	1	1	2	1.3
Clavicle	1	0	1	0.6
Acromion	3	0	3	1.9
Humerus	11	1	12	7.8
Radius	15	2	17	11.0
Ulna	23	2	25	16.2
Lunate	1	0	1	0.6
Scaphoid	3	0	3	1.9
Trapezium	1	0	1	0.6
Metacarpal	18	1	19	12.3
Phalanx (hand)	16	8	24	15.5
Sacral spine	1	0	1	0.6
Femur	1	0	1	0.6
Patella	6	0	6	3.9
Tibia	13	1	14	9.1
Fibula	7	1	8	5.2
Cuboid	1	0	1	0.6
Metatarsal	1	0	1	0.6

**Table 3:** Associated tendon and neurovascular machete cut injuries.

Associated injuries	Number of patients (%)
Extensor tendon hand injury	19 (20.9)
Flexor tendon hand injury	10 (10.9)
Patella tendon injury	2 (2.2)
Ulna nerve injury	5 (5.5)
Radial nerve injury	5 (5.5)
Median nerve injury	2 (2.2)
Peroneal nerve injury	2 (2.2)
Radial artery injury	5 (5.5)
Ulna artery injury	3 (3.3)
Digital artery injury	2 (2.2)
Anterior tibia artery injury	1 (1.1)

The mean interval between fracture and presentation to the hospital was 18 ± 22.0hrs.Sixty five (71.4%) patients presented within the first 6 h of fracture whereas 26 (28.6%) patients presented later than 6 h from the time of injury. Seventeen (34.7%) of the patient whose fractures occurred in rural setting and 7(16.7%) of the patients with fractures in that occurred urban areas arrived the hospital later than 6hrs of injury but this difference in the rate of delayed and later presentation was not significant (p = 0.052).Sixty two (68.1%) of the patient had pre-hospital care, and the rest 39.1% had no pre hospital care.

**Figure 2:**
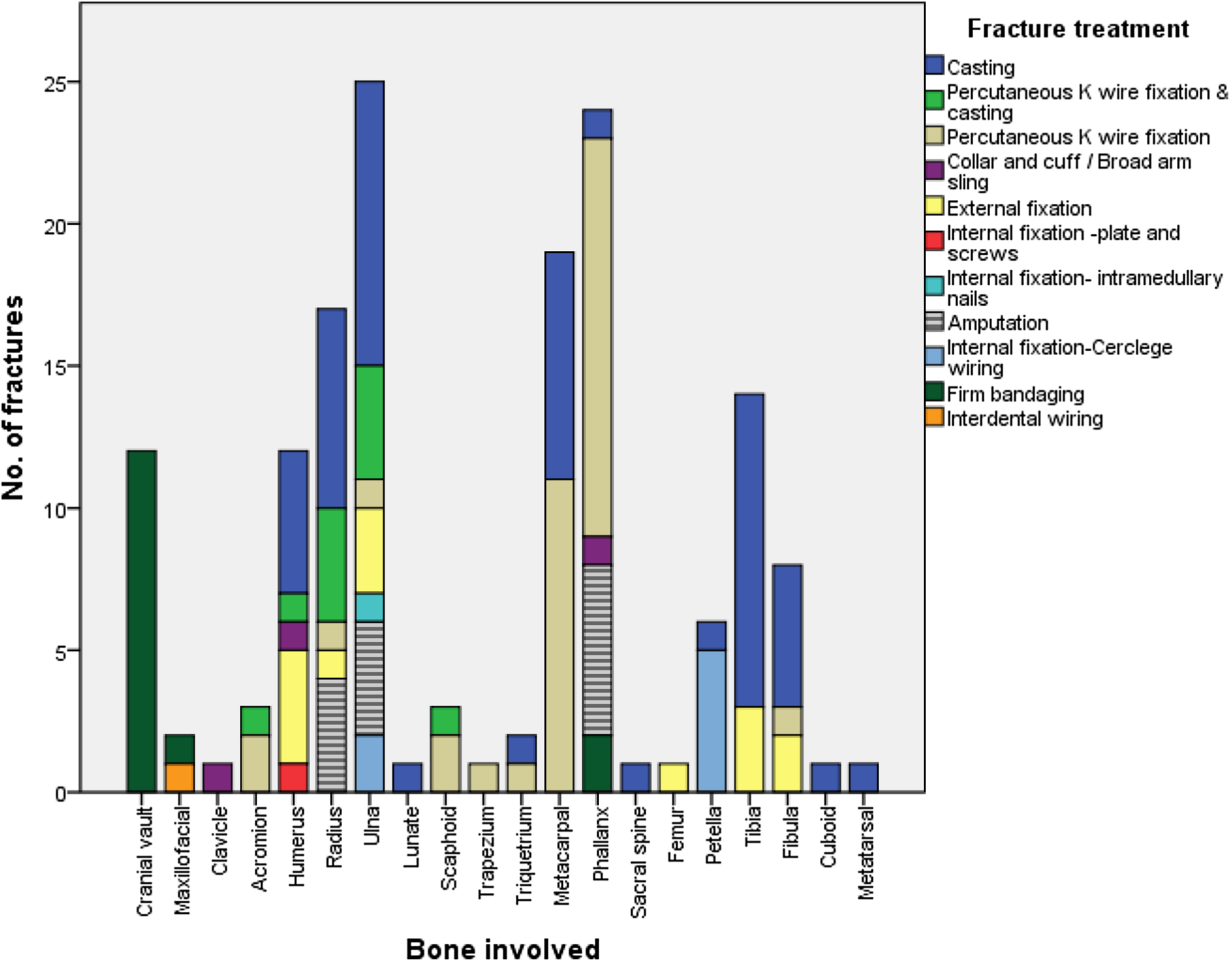
Distribution of fracture treatment by bone involved. Figure designed using IBM Statistical package for Social Sciences, IBM SPSS Statistics version 20 (IBM Corp. Armonk, N.Y. USA) statistical soft ware https://www.ibm.com/products/spss-statistics.

Of the 154 open fractures, the definitive wound care involved primary closure (56, 36.4%), delayed primary closure (88, 57.1%), secondary wound closure (7, 4.5%) and split skin grafting (1, 0.6%). One patient with open fractures in two bones self discharged against advice without definitive wound care. Fig. 2 shows that casting was the topmost form of definitive treatment for radius, ulna, tibia and fibula fractures whereas cranial vault fractures where managed conservatively with firm bandaging after closure of overlying scalp wound (2 of the patients with cranial vault fracture were transferred to another hospital for operative neurosurgical intervention).

Anemia, wound infection and hemorrhagic shock were the three top complications as shown in Table 4. Twenty eight (30.8%) of the patients received blood transfusion. Of the 154 fractures, wound infection was observed in 50 (32.5%) fractures. The rate of wound infection was significantly related to anatomical region of the fractured bone, the infection rate was 16.7% for fractures in head region and 27.8% and 63.0% for fracture wounds in upper and lower extremities respectively (p = 0.002). The incidence of wound infection also correlated with injury hospital arrival interval, a higher rate was observed in presentations later than 6 h compared to first 6 h of injury (68.2% vs. 18.2%, p < 0.001).

**Table 4:** Complications of machete cut fractures (N = 91patients).

Complications	Number of patients (%)
Anaemia	34 (37.4)
Wound infection	26 (28.6)
Hypovolaemic shock	14 (15.4)
Extremity gangrene	4 (4.4)
Residual Nerve palsy	4 (4.4)
Residual Joint stiffness	4 (4.4)
Malunion	1 (1.1)
Delayed union	1 (1.1)
Hemi paresis	1 (1.1)
Deltoid muscle atrophy	1 (1.1)

The mean and median duration of admission was 18.1 days and 12 days respectively. Prolonged duration of hospital admission correlated significantly with injury to hospital arrival interval later than 6 h (p < 0.001) and to wound infection (p < 0.001) as shown in Table 5.

**Table 5:** Duration of hospital admission by demographics, intervention related factor and complications.

	Duration of Admission (days)			Total (%)	*p* value
	1–7 N (%)	8–14 N (%)	> 14 N (%)		
**Age (year)**					
0–17	4 (50.0)	0 (0.0)	4 (50.0)	8 (8.8)	0.766
18–39	25 (39.5)	12 (19.0)	26 (41.3)	63 (69.2)	
40–65	6(42.9)	4 (28.6)	4 (26.6)	14 (15.4)	
> 65	2 (33.3)	1 (16.7)	3 (50.0)	6 (6.6)	
**Sex**					
Female	1 (12.5)	4 (50.0)	3 (37.5)	8 (8.8)	0.042
Male	36 (43.4)	13 (15.7)	34 (41.0)	83(91.2)	
**Injury to hospital interval (h)**					
0–6	31 (47.7)	16 (24.6)	18 (27.7)	65 (71.4)	< 0.001
> 6	6 (23.1)	1 (3.8)	19 (73.1)	26 (28.6)	
**Anaemia**					
Yes	11 (32.4)	4 (11.8)	19 (55.9)	34 (37.4)	0.067
No	26 (45.6)	13 (22.8)	18 (22.8)	57 (62.6)	
**Hypovolemic shock**					
Yes	6 (42.9)	3 (21.4)	5 (35.7)	14 (15.4)	0.911
No	31 (40.3)	14 (18.2)	32 (41.6)	77 (84.6)	
**Wound infection**					
Yes	2 (7.7)	4 (15.4)	20 (76.9)	26 (28.6)	< 0.001
No	35 (53.8)	13 (20.0)	17 (26.2)	65 (71.4)	

Of the 154 fractures, 9 (5.8%) ended in amputation and the outcome of fracture healing in 27 (17.5%) was unknown among those that died while on hospital admission, transferred or self discharged against medical advice. Of the 118 fractures that were treated and followed up to a minimum of one year, normal fracture union (116, 98.3%), mal union (1, 0.6%) and delayed union (1, 0.6%) were observed.

Seventy three (80.2%) of the patients recovered fully; 11 (12.1%) patients discharged self against advice, and 5 (5.5%) were transferred. Two patients died giving a case fatality rate of 2.2%. None of the patients that died received pre hospital care and the cause of death in each of them was hemorrhagic shock.

## Discussion

Machete cut fracture is an important component of machete related injuries in our environment. The results of this study indicate that the victims of machete cut fractures were predominantly young active males. This is not surprising because previous published report on machete related injuries in a similar setting indicate preponderance of the same age and gender category^1^. The preponderance of the young and active segment of the population and its associated negative socio-economic impact by implication compounds the burden of machete cut fractures in this setting. In this study, the highest rate of accidental machete cut fracture observed among children suggest higher vulnerability of this age group to accident related machete injuries especially in farming related activities. This calls for proper machete technique and more supervision of this age category during farming activities that involves the use of machete.

Fractures including machete related ones are considered grievous hurt from the medico legal point of view^13^. A published report also indicates that homicidal machete wounds are characteristically multiple and commonly situated over head and neck region with associated defense wound in upper extremity^14^. In this study, the proportion of the patients that were multiply fractured, sustained additional distant machete soft tissue injuries (60%) or had head and upper extremity machete wound all indicate homicidal intent of injury in significant number of these patients. The involvement of interpersonal violence with homicidal intent in a significant proportion of the victims might be under-recognized important aspect of machete cut fractures. This calls for measures aimed at reducing interpersonal violence to barest minimum.

In this study the percentage of partial fractures was almost close to14% reported by Serra et.al^7^ for forearm machete cut fractures. Communited fracture is common in this study and in the series reported by Serra et.al^7^. However, these findings are different from rarity of communited fractures in the series reported by Rymaszewski and Caullay^3^. The reason for the variations in the rates of communited fracture is not evident. In a recent published report, Cohen et al. demonstrated an association between fracture line morphology and impact velocity on the bone, and that a moderate—high energy impact results in communited fracture^15^. Thus, the types of fracture line morphology observed indicate variation in the impact velocity of machete cut on the fractured bones. Furthermore, the result of this study indicate there is no remote possibility of high impact velocity fracture although machete cut often results in sharp division of tissues and focal transfer of energy. This should be taken into consideration in evaluation of the injury and treatment strategies.

Primary wound closure in a third of the fractures and casting as the topmost definitive treatment of the fractures involving long bones in this series, where about 70 percent of the patients presented within 6 h of injury, compared to osteosynthesis for all the cases reported by Serra et al^7^ and Rymaszewski and Caullay^3^ suggest, among other reasons, the attending surgeons were more on the conservative side in dealing with machete related open fractures or a more prevalence of severely contaminated wound in this setting.

The result of this study indicates that associated tendon and neurovascular injuries, which have implications in the overall outcome of treatment, is quite common in machete cut fracture. This is not surprising because division of soft tissues is expected as a wielded machete slices through it to lacerate a bone. Thus, the importance of thorough examination to rule out associated tendon and neurovascular injury cannot be over emphasized.

The overall wound infection rate in this study is within the range of overall incidence of wound infection (0–50%) after open fractures in published reports^16,17^. However, this rate and the infection rate in upper extremity fractures observed are higher than 14.3% reported by Serra et.al^7^ for forearm machete cut fractures. Apart from presentation to the hospital later than 6hours, which was significantly associated with this high wound infection rate, other factors for the relatively high wound infection rate in this study are not evident.

The proportion of the patients that had no pre-hospital care was relatively high. A pre-hospital care could have prevented the two cases of mortality from haemorrhagic shock as well as high incidence of anaemia occasioned by massive blood loss and hemorrhagic shock observed in this study. This calls for public enlightenment on simple first aid measures to contain blood loss following open injuries such as machete cut in this setting.

The duration of hospital admission is directly related to morbidity and cost of care in machete cut fractures. However, the result of this study as shown in Table 5 indicates that measures aimed at early presentation of the victims to the hospital for prompt intervention, and prevention of wound infection are needed to reduce the duration of hospital admission, and by implication the morbidity rate.

The limitation of this study is in being hospital based one and the data obtained may not be a representation of entire population. However, the finding in this study can be used as base line data in future study.

## Conclusion

In our environment, open fractures from machete cut of varying levels of impact energy occur mainly in physical violence affecting predominantly the young age group, and are more prevalent in dry season. These calls for preventive mechanisms, which should include educational campaign directed towards young people and intensified during the dry season. Although the rate of normal union of the fractures was over ninety eight percent the pattern of morbidity and mortality observed calls for measures to improve the rates of early presentation of patients to hospital, and pre hospital care.
